# Supplementary material for: Trophic resources of the edaphic microarthropods: A worldwide review of the empirical evidence
Source: Heliyon. 2023 Sep 28;9(10):e20439. doi: 10.1016/j.heliyon.2023.e20439 (PMC10560771; doi:10.1016/j.heliyon.2023.e20439)
Supplement: MMC 3 — Electronic Supplementary Material III (ESM III): Flowchart showing the criteria used in the bibliographic search and selection. [file mmc3.pdf]

Supplementary material III: Bibliography search and paper selection process flowchart

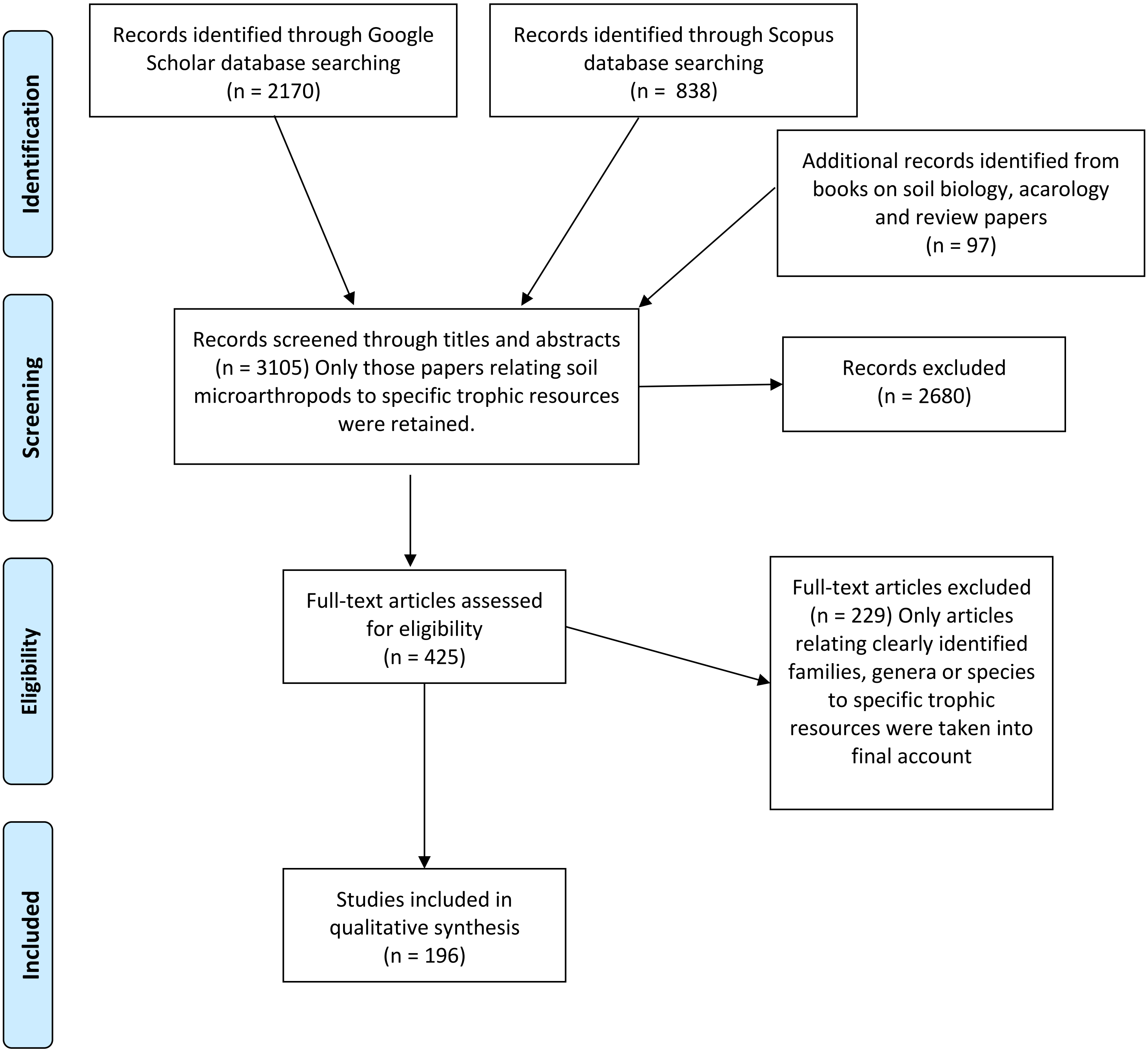

From: Moher D, Liberati A, Tetzlaff J, Altman DG, The PRISMA Group (2009). *P*referred *R*eporting *I*tems for *S*ystematic Reviews and *M*eta-*A*nalyses: The PRISMA Statement. PLoS Med 6(7): e1000097. doi:10.1371/journal.pmed1000097

For more information, visit www.prisma-statement.org.
